# Thermal damage in three-dimensional vivo bio-tissues induced by moving heat sources in laser therapy

**DOI:** 10.1038/s41598-019-47435-7

**Published:** 2019-07-29

**Authors:** Jingxuan Ma, Xianfeng Yang, Yuxin Sun, Jialing Yang

**Affiliations:** Institute of Solid Mechanics, School of Aeronautic Science and Engineering, Beihang University, Beijing, 100191 P.R. China

**Keywords:** Skin models, Thermodynamics

## Abstract

The thermal damage of a three-dimensional bio-tissue model irradiated by a movable laser beam was studied in this work. By employing the DPL biological heat conduction model and Henriques’ thermal damage assessment model, the distribution of burn damage of *vivo* human tissue during laser therapy was analytically obtained. The influences of laser moving velocity, laser spot size, phase lags of heat flux and temperature gradient were discussed. It was found that the laser moving speed and the laser spot size greatly influence the thermal damage degree by affecting the energy concentration degree. The increases of the laser moving speed and laser spot size can enlarge the irradiated region and reduce the burn degree. A greater phase lag of temperature gradient led to lower accumulation of thermal energy and lower burn degree. However, the increment of heat flux phase lag leads to the thermal energy accumulation and more serious burn degree in the irradiated region.

## Introduction

The thermotherapy is a useful approach in medical field, such as hyperthermia, laser soldering, laser ablation, laser surgery and other thermal treatment methods^1^. Many researchers have explored the pathological mechanism and thermal behavior in different laser surgeries. For example, Tuncer *et al*.^2^ declared the advantages of the CO_2_ laser surgery in oral soft tissue pathologies, such as minimal side effects, good pain control, minimally invasive procedure and favorable prognosis. Semenyuk^3^ explored the laser-induced thermal behavior of retinal pigmented epithelium and the adjacent layers in a human eye, in order to specify laser optimal parameters and protect the normal tissue in an ophthalmic surgery.

In order to maximize the therapeutic efficiency while ensuring the patients’ safety, the thermal damage induced by the laser beam need to be predicted precisely. The accurate description of the bio-heat conduction process in living tissues is primary to predict the thermal behavior. Pennes^4^ was the first to develop a biological heat conduction model to describe the thermal transfer process in the *vivo* tissue. The Pennes’ model has been employed as an efficient model in a lot of researches on bio-heat transfer. Among them, Ma *et al*.^5^ investigated the thermal response in the laminated bio-tissue during the nanoshell assisted laser hyperthermia for subcutaneous tumor. Shih *et al*.^6^ presented a theoretical analysis of the *Pennes* bioheat transfer equation with sinusoidal heat flux condition on skin surface.

However, the Fourier’s law assumed that the heat propagation speed is infinite, which was not precise in transient thermal conduction. In order to describe the thermal transfer process in living bio-tissues more accurately, the non-Fourier effects should be considered. So many researchers made modifications on the Pennes’ model by introducing the non-Fourier effects. Among these, two models are commonly used at present. The first one is a hyperbolic heat conduction model, which is developed by Cattnaeo^7^ and Vernotte^8^. This model introduced a heat flux relaxation time to describe the thermal transfer process with a finite velocity, and is referred to as C-V model hereinafter. Zhang *et al*.^9^ proposed a thermomechanical model for multilayered structure subjected to heat deposition with the thermal barrier under consideration. Tzou^10^ improved the C-V model by considering the phase lags of both heat flux and temperature gradient and proposed a dual-phase-lag heat conduction model (DPL model). Ma *et al*.^11^ discussed the combined effects of these phase lags and moving thermal source on a square plate. Kumar *et al*.^12^ theoretically investigates the thermal behavior in a living biological tissue under various coordinate systems and different non-Fourier boundary conditions with the dual-phase-lag bioheat transfer model during thermal therapy. Liu *et al*.^13^ simultaneously and inversely estimated the values of the relaxation times and heat conductivity based on the DPL model with the measurement date in the literature.

The patients’ safety should come first during the laser treatment procedure, so it is important to study the bio-tissue thermal damage and there have been numbers of researches on this topic. Ibarguren *et al*.^14^ experimentally conducted the thermal response of soft tissue subjected to different laser beams. It was found that the Er, Cr:YSGG laser with water/air spray causes the least serious degree of thermal damage. Zhou *et al*.^15^ numerically investigated the thermal damage in laser-irradiated biological tissues by using the DPL bioheat conduction model. Li *et al*.^16^ studied thermal damage in thermal ablation with the thermal-mechanical effect of soft tissues under consideration. The thermal-mechanical behaviors of soft tissues was described through non-rigid motion dynamics. Paul *et al*.^17^ employed the finite element method to calculate the thermal response of bio-tissue embedded with large blood vessels during photo-thermal heating process. Zhou *et al*.^18^ explored the thermal damage in the human tissue by using both C-V model and Pennes model. The comparison between the results obtained by the two models proved that the non-Fourier effects brought significant influence on the burn damage in biological tissue. Liu and Wang^19^ estimated thermal damage in laser-irradiated biological tissue by using DPL model. The influences of blood perfusion and metabolic heat generation on the temperature field and thermal damage were discussed. Afrin and Zhang^20^ studied the effects of uncertainties of laser exposure time, phase lag times, blood perfusion coefficient, scattering coefficient and diffuse reflectance of light on the burn damage of *vivo* biological tissue by laser irradiation.

The non-Fourier effects and a movable laser source can be found in the clinical laser surgeries, so the assessment of thermal damage induced by moving sources should be concerned. Yet few literatures focus on the burn damage caused by a movable laser beam on the basis of DPL bio-heat conduction model. To address this issue, the burn procedure in the three-dimensional *vivo* human tissue which is induced by a movable laser beam is explored in this study. The effects of blood vessel capillaries and metabolic heat generation are under consideration and the DPL model was employed. The mode superposition method and variable-separation method are used to derive the theoretical solutions for the governing equations. The burn damage intensity of the bio-tissue is determined and the influences of laser spot size, phase lags and laser moving velocity are discussed.

## Mathematical Models

### Heat conduction model

The DPL model is employed to describe the thermal transfer procedure, which is modified from the Fourier’s law with two relaxation times under consideration. The DPL model can be described by the following formulation^21^,

1 $$q(\overrightarrow{r},t+{\tau }_{q})=-\,k\nabla T(\overrightarrow{r},t+{\tau }_{T})$$

where *t*, *T*, *q*, $\overrightarrow{r}$ and *k* represent the time, the temperature of the tissue, the heat flux, the position vector and the heat conductivity, respectively. ${\tau }_{q}$ is the heat flux phase lag and ${\tau }_{T}$ represents the temperature gradient phase lag.

According to the DPL model, the heat conduction equation can be expressed as:

2 $${\nabla }^{2}(T+{\tau }_{T}\frac{\partial T}{\partial t})=\frac{1}{\alpha }(\frac{\partial T}{\partial t}+{\tau }_{q}\frac{{\partial }^{2}T}{\partial {t}^{2}})$$

where *α* is the thermal diffusivity.

### Bio-heat transfer model of living tissue

The heat conduction process in living tissue is significantly complicated because of the involvement of the heat conduction between blood and tissues, blood perfusion in vascular beds and metabolic heat generation. With the non-Fourier effects under consideration, an effective bio-heat transfer model that includes effects of the capillary vessel systems, the metabolism and the relaxation time of temperature gradient and heat flux can be expressed as^22^:

3 $$\begin{array}{l}\rho C{\tau }_{q}\frac{{\partial }^{2}T}{\partial {t}^{2}}+(\rho C+{\tau }_{q}{w}_{b}{\rho }_{b}{C}_{b})\frac{\partial T}{\partial t}\\ \begin{array}{rcl} & = & k{\nabla }^{2}T+k{\tau }_{T}\frac{\partial }{\partial t}({\nabla }^{2}T)+{w}_{b}{\rho }_{b}{C}_{b}({T}_{a}-T)\\  &  & +\,{\tau }_{q}(\frac{\partial {Q}_{m}}{\partial t}+\frac{\partial Q}{\partial t})+{Q}_{m}+Q\end{array}\end{array}$$

where, $\rho$ and ${\rho }_{b}$ represent the density of the human tissue and the blood. *C* and *C*_*b*_ are specific heat of the *vivo* tissue and blood. *w*_*b*_ is the blood perfusion in the human tissue. *T*_*a*_ represents the human core temperature. *Q*_*m*_ represents heat production rate caused by metabolism and *Q* is energy power of external source.

### Energy absorption model

In the above equation, the external heat source *Q* can be written as^23^:

4 $$Q(\overrightarrow{r},t)={\mu }_{t}\phi (\overrightarrow{r},t)$$

where, *μ*_*t*_ and $\phi (\overrightarrow{r},t)$ are the absorption coefficient of material and the local energy distribution, respectively. For the three-dimensional *vivo* human tissues, $\phi (\overrightarrow{r},t)$ can be expressed in the following form:

5 $$\phi (\overrightarrow{r},t)=(1-R)I(t)\varphi (x,y)\,\exp \,(\,-\,{\mu }_{t}z)$$

where, *R* represents the optical reflection ratio of the human tissue. *I*(*t*) is the power density of laser source. *μ*_*t*_ represents the optical attenuation coefficient, and $\varphi (x,y)$ is the energy distribution on the surface.

### Thermal damage model

The burn damage caused by a thermal source on a living tissue is complex and multidisciplinary, which depends on the power of the heat source and its duration. Henriques and Moritz^24^ proposed an expression for the denaturation process based on the first order approximation of the Arrhenius equation. The denaturation rate *K* can be written as:

6 $$K(T)=A\,\exp \,(-\frac{{E}_{a}}{RT})$$

where, *A* is the tissue frequency factor, *R* the universal gas constant and *E*_*a*_ the activation energy of the denaturation reaction. The evaluation parameter of burn damage can be expressed as:

7 $${\rm{\Omega }}(t)={\int }_{0}^{t}\,A\,\exp \,(-\frac{{E}_{a}}{RT})dt$$

### Governing equation of the problem

**Figure 1:**
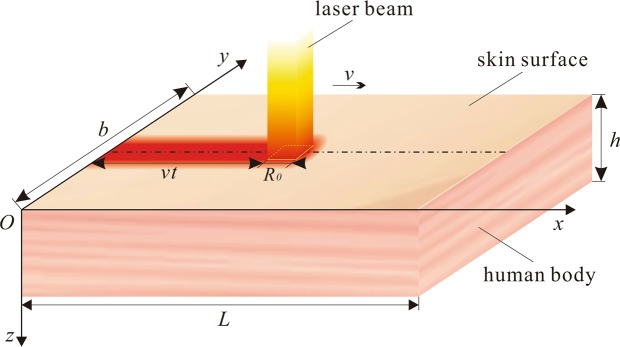
Illustration of the human tissue subjected to a movable laser source.

The human tissue is modelled as a cuboid as shown in Fig. 1. The parameters *L*, *b* and *h* represent the length, width and height of the cuboid tissue, respectively. The top surface of the human tissue is irradiated by a square laser spot, which moves along the middle axis with velocity *v*.

The governing equation based on DPL model can be written as:

8 $$\begin{array}{l}\rho C{\tau }_{q}\frac{{\partial }^{2}\theta }{\partial {t}^{2}}+(\rho C+{\tau }_{q}{w}_{b}{\rho }_{b}{C}_{b})\frac{\partial \theta }{\partial t}\\ \begin{array}{rcl} & = & k{\nabla }^{2}\theta +k{\tau }_{T}\frac{\partial }{\partial t}({\nabla }^{2}\theta )-{w}_{b}{\rho }_{b}{C}_{b}\theta \\  &  & +\,{\tau }_{q}(\frac{\partial {Q}_{m}}{\partial t}+\frac{\partial {Q}_{l}}{\partial t})+{Q}_{m}+{Q}_{l}\end{array}\end{array}$$

where *Q*_*l*_ represents the laser heating source, $\theta =T-{T}_{a}$ the temperature rise, ${T}_{a}=37\,^\circ {\rm{C}}$ the temperature of the human body. The heat energy of the laser beam in this study is treated as a body heat source in the tissue model and the expression can be written as:

9 $${Q}_{l}={\mu }_{t}\phi (z){Q}_{1}(x){Q}_{2}(y)$$

where, $\phi (z)=(1-R){I}_{0}\,\exp \,(\,-\,{\mu }_{t}z)$ represents the distribution in laser irradiation direction for the high-absorption tissues. *Q*_1_(*x*) and *Q*_2_(*y*) are the spatial distribution functions of the laser power density which can be expressed as:

10 $${Q}_{1}(x)=H(x-vt)-H[x-(vt+{R}_{0})]$$

11 $${Q}_{2}(y)=H[y-(\frac{b}{2}-\frac{{R}_{0}}{2})]-H[y-(\frac{b}{2}+\frac{{R}_{0}}{2})]$$

where, *H*(*x*) is the Heaviside function, and *R*_0_ is the laser spot measure.

Considering this living tissue model as a part of human body, the temperatures on the side-faces and bottom surface are set as *T*_*a*_ and the top surface is adiabatic. So the boundary conditions can be written as:

12 $$\{\begin{array}{c}{\theta |}_{x=0}={\theta |}_{x=l}=0\\ {\theta |}_{y=0}={\theta |}_{y=b}=0\\ {\theta |}_{z=h}=0\\ {\frac{\partial \theta }{\partial z}|}_{z=0}=0\end{array}$$

The initial conditions can be expressed as:

13 $$\{\begin{array}{c}{\theta |}_{t=0}=0\\ {\frac{\partial \theta }{\partial t}|}_{t=0}=0\end{array}$$

## Solution for Governing Equation

The governing equation (8) can be solved by using the variables separation method and the mode superposition method. The solution of equation (8) can be written as following^25^:

14 $$\theta (x,y,z,t)=\sum _{m=1}^{\infty }\,\sum _{n=1}^{\infty }\,\sum _{s=0}^{\infty }\,{T}_{mns}(t){X}_{m}(x){Y}_{n}(y){Z}_{s}(z)$$

where, *X*_*m*_(*x*), *Y*_*n*_(*y*) and *Z*_*s*_(*z*) are the mode functions, which are expressed as

15 $$\{\begin{array}{rcl}{X}_{m}(x) & = & \sin \,({\beta }_{m}x)\\ {Y}_{n}(y) & = & \sin \,({\gamma }_{n}y)\\ {Z}_{s}(z) & = & \cos \,({\mu }_{s}z)\end{array}$$

and *β*_*m*_, *γ*_*n*_ and *μ*_*s*_ are eigenvalues, which are given by

16 $$\{\begin{array}{ll}{\beta }_{m}=m\pi , & m=1,2,3\ldots \\ {\gamma }_{n}=\frac{L}{b}n\pi , & n=1,2,3\ldots \\ {\mu }_{s}=\frac{L}{h}\frac{(2s+1)}{2}\pi , & s=0,1,2\ldots \end{array}$$

Substitute equation (14) into equation (8), and ${T}_{mns}(t)$ can be obtained as:

17 $${T}_{mns}(t)={T}_{mns}^{(0)}(t)+{T}_{mns}^{\ast }(t)$$

By introducing the following parameters

18 $$\{\begin{array}{l}{A}_{1}={\tau }_{q}\\ {A}_{2}=1+{\tau }_{q}{\overline{w}}_{b}+{\lambda }_{mns}^{2}{\tau }_{T}\\ {A}_{3}={\lambda }_{mns}^{2}+{\overline{w}}_{b}\end{array}$$

where, $${\lambda }_{mns}^{2}={\beta }_{m}^{2}+{\gamma }_{n}^{2}+{\mu }_{s}^{2}$$ and $${\overline{w}}_{b}=\frac{{w}_{b}{\rho }_{b}{C}_{b}}{\rho C}$$.

The solution of ${T}_{mns}^{(0)}(t)$ can be derived as the following:

If $${\rm{\Delta }}={A}_{2}^{2}-4{A}_{1}{A}_{3} > 0$$,

19 $${T}_{mns}^{(0)}(t)={C}_{1}\,\exp \,(-\frac{1}{2}\frac{({A}_{2}+\sqrt{{\rm{\Delta }}})}{{A}_{1}}t)+{C}_{2}\,\exp \,(-\frac{1}{2}\frac{({A}_{2}-\sqrt{{\rm{\Delta }}})}{{A}_{1}}t)$$

where,

20 $$\begin{array}{rcl}{C}_{1} & = & -\frac{1}{2\sqrt{{\rm{\Delta }}}}\{\begin{array}{c}\sin ({\beta }_{m}{R}_{0})[2{F}_{c}{A}_{1}v{\beta }_{m}+{F}_{s}({A}_{2}-\sqrt{{\rm{\Delta }}})]\\ +\,[\cos ({\beta }_{m}{R}_{0})-1][2{F}_{s}{A}_{1}v{\beta }_{m}+{F}_{c}(\sqrt{{\rm{\Delta }}}-{A}_{2})]\end{array}\}\\  &  & -\,\frac{{g}_{1}(\sqrt{{\rm{\Delta }}}-{A}_{2})}{2\sqrt{{\rm{\Delta }}}{A}_{3}}\\ {C}_{2} & = & \frac{1}{2\sqrt{{\rm{\Delta }}}}\{\begin{array}{c}\sin ({\beta }_{m}{R}_{0})[2{F}_{c}{A}_{1}v{\beta }_{m}+{F}_{s}({A}_{2}+\sqrt{{\rm{\Delta }}})]\\ +\,[\cos ({\beta }_{m}{R}_{0})-1][2{F}_{s}{A}_{1}v{\beta }_{m}+{F}_{c}(-\sqrt{{\rm{\Delta }}}-{A}_{2})]\end{array}\}\\  &  & -\,\frac{{g}_{1}(\sqrt{{\rm{\Delta }}}+{A}_{2})}{2\sqrt{{\rm{\Delta }}}{A}_{3}}\end{array}$$

If $${\rm{\Delta }}={A}_{2}^{2}-4{A}_{1}{A}_{3} < 0$$,

21 $${T}_{mns}^{(0)}(t)={C}_{1}\,\exp \,(\,-\,\frac{1}{2}\frac{{A}_{2}}{{A}_{1}}t)\,\cos \,(\frac{\sqrt{\,-\,{\rm{\Delta }}}}{2{A}_{1}}t)+{C}_{2}\,\exp \,(\,-\,\frac{1}{2}\frac{{A}_{2}}{{A}_{1}}t)\,\sin \,(\frac{\sqrt{\,-\,{\rm{\Delta }}}}{2{A}_{1}}t)$$

where,

22 $$\begin{array}{rcl}{C}_{1} & = & -{F}_{c}\,\cos \,({\beta }_{m}{R}_{0})+{F}_{s}\,\sin \,({\beta }_{m}{R}_{0})+{F}_{c}-\frac{{g}_{1}}{{A}_{3}}\\ {C}_{2} & = & \frac{1}{\sqrt{-{\rm{\Delta }}}}\{\begin{array}{c}\sin ({\beta }_{m}{R}_{0})(2{F}_{c}{\beta }_{m}v{A}_{1}+{A}_{2}{F}_{s})\\ +\,[\cos ({\beta }_{m}{R}_{0})-1](2{F}_{s}{\beta }_{m}v{A}_{1}-{A}_{2}{F}_{c})\end{array}\}-\frac{{A}_{2}{g}_{1}}{\sqrt{-{\rm{\Delta }}}{A}_{3}}\end{array}$$

If $${\rm{\Delta }}={A}_{2}^{2}-4{A}_{1}{A}_{3}=0$$,

23 $${T}_{mns}^{(0)}(t)={C}_{1}\,\exp \,(-\frac{{A}_{2}}{2{A}_{1}}t)+{C}_{2}t\,\exp \,(-\frac{{A}_{2}}{2{A}_{1}}t)$$

where,

24 $$\begin{array}{rcl}{C}_{1} & = & -{F}_{c}\,\cos \,({\beta }_{m}{R}_{0})+{F}_{s}\,\sin \,({\beta }_{m}{R}_{0})+{F}_{c}-\frac{{g}_{1}}{{A}_{3}}\\ {C}_{2} & = & \frac{1}{2{A}_{1}}\{\begin{array}{c}\sin ({\beta }_{m}{R}_{0})(2{F}_{c}{\beta }_{m}v{A}_{1}+{A}_{2}{F}_{s})\\ +\,[\cos ({\beta }_{m}{R}_{0})-1](2{F}_{s}{\beta }_{m}v{A}_{1}-{A}_{2}{F}_{c})\end{array}\}-\frac{{A}_{2}{g}_{1}}{2{A}_{1}{A}_{3}}\end{array}$$

While ${T}_{mns}^{\ast }(t)$ can be obtained as the following:

25 $$\begin{array}{rcl}{T}_{mns}^{\ast }(t) & = & {F}_{c}\{\cos \,[{\beta }_{m}(v\tau +{R}_{0})]-\,\cos \,({\beta }_{m}vt)\}\\  &  & +\,{F}_{s}\{\sin \,({\beta }_{m}vt)-\,\sin \,[{\beta }_{m}(vt+{R}_{0})]\}+\frac{{g}_{1}}{{A}_{3}}\end{array}$$

where,

26 $${F}_{c}=\frac{{g}_{2}({A}_{1}{v}^{2}{\beta }_{m}^{2}-{A}_{3})-{g}_{3}{A}_{2}v{\beta }_{m}^{2}}{{\beta }_{m}[{A}_{1}^{2}{v}^{4}{\beta }_{m}^{4}+({A}_{2}^{2}-2{A}_{1}{A}_{3}){v}^{2}{\beta }_{m}^{2}+{A}_{3}^{2}]}$$

27 $${F}_{s}=\frac{{g}_{2}{A}_{2}v{\beta }_{m}+{g}_{3}{\beta }_{m}({A}_{1}{v}^{2}{\beta }_{m}^{2}-{A}_{3})}{{\beta }_{m}[{A}_{1}^{2}{v}^{4}{\beta }_{m}^{4}+({A}_{2}^{2}-2{A}_{1}{A}_{3}){v}^{2}{\beta }_{m}^{2}+{A}_{3}^{2}]}$$

28 $${g}_{1}=\frac{8{\overline{Q}}_{m}}{\rho C}[\frac{1+{(-1)}^{m+1}}{m\pi }]\,[\frac{1+{(-1)}^{n+1}}{n\pi }]\,[\frac{2{(-1)}^{s}}{\pi (2s+1)}]$$

29 $${g}_{2}=\frac{8{q}_{0}}{Lbh}\frac{2b\,\sin \,(\frac{2\pi {R}_{0}}{2b})\,\sin \,(\frac{n\pi }{2})}{n\pi }\frac{4{\mu }_{t}{h}^{2}+2h\pi (2s+1)\,{(-1)}^{n}\,\exp \,(-{\mu }_{t}h)}{4{h}^{2}{\mu }_{t}^{2}+4{\pi }^{2}{s}^{2}+4{\pi }^{2}s+{\pi }^{2}}$$

30 $${g}_{3}={g}_{2}{\tau }_{q}v$$

31 $${q}_{0}=\frac{{\mu }_{t}{I}_{0}(1-R)}{\rho C}$$

Finally, substituting the temperature distribution formulations into equation (7) yields the burn damage in the human tissue.

## Physical Parameters

Table 1 gives the parameters of the laser source used in this problem. Table 2 shows the thermo-physical parameters of the human tissue^26^.

**Table 1:** Parameters of the laser source.

Parameters	Values
*I*_*0*_, Power density of laser source (W/m^3^)	1 × 10^6^
*R*, optical reflection ratio	0.024
*R*_*0*_, radius parameter of laser beam (m)	2 × 10^−3^

**Table 2:** Thermo-physical parameters of the human tissue.

Parameters	Blood	Human tissue
Specific heat (J/kg∙K)	3770	3600
Density (kg/m^3^)	1060	1190
Thermal conductivity (W/m∙K)	—	0.235
Metabolic heat Generation (W/m^3^)	—	368.1
Length (m)	—	0.018
Width (m)		0.018
Thickness (m)		0.009
Blood perfusion (s^−1^)	—	1.87
*μ*_*t*_, absorption coefficient (m^−1^)	—	2000

The parameters for burn evaluation, which are introduced in equations (6) and (7), are shown in Table 3 ^26^. It has been accepted that: ${\rm{\Omega }}=0.53$ represents the first-degree burn, ${\rm{\Omega }}=1$ for the second degree-burn and ${\rm{\Omega }}={10}^{4}$ for the third-degree burn^24,26,27^.

**Table 3:** Parameters for thermal damage prediction.

Temperature rage (^o^C)	*E*_*α*_/*R* (K)	*A*, Frequency factor (s^−1^)
*T* ≤ 55	7.5 × 10^4^	3.1 × 10^98^
*T* > 55	3.54 × 10^4^	5.0 × 10^45^

## Results and Discussions

**Figure 2:**
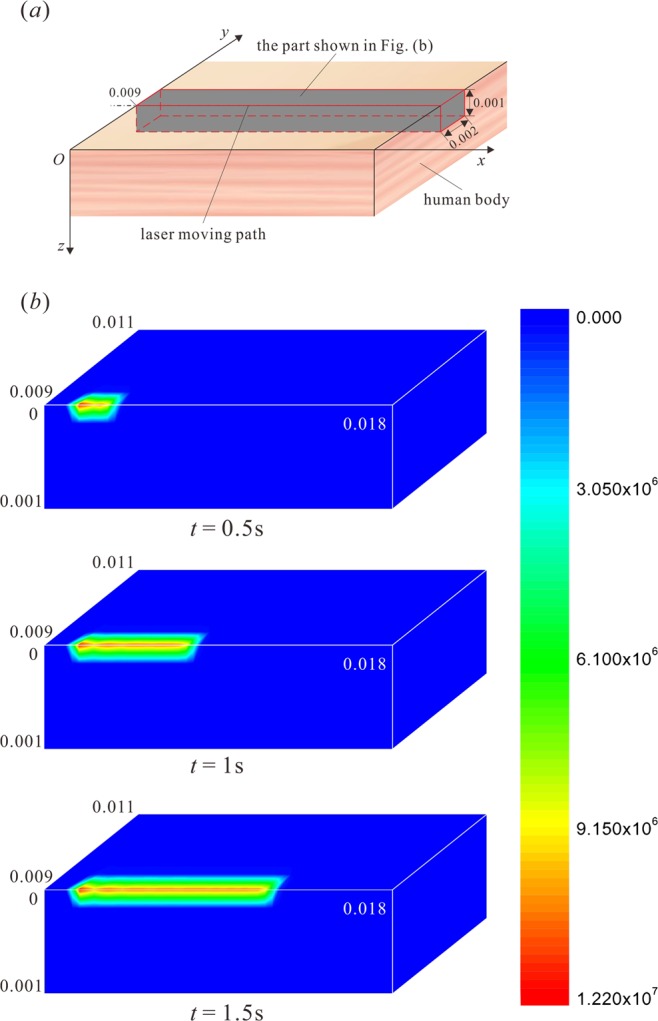
Distribution of thermal damage in the target tissue. (**a**) The sketch of the selected part for the exhibition of thermal damage distribution; (**b**) Empyrosis distribution in the heat-affected zone.

The empyrosis distribution in the bio-tissue due to a moving laser is derived on the basis of the analytical solution of temperature response, which is introduced in Section 3. The thermal conductivity of bio-tissue is small, so the burn mainly occurs in a small vicinity region of the irradiated area where the laser energy is concentrated. Firstly, the empyrosis distribution in an appropriate part of the target tissue (the grey part in Fig. 2(a)) is exhibited in Fig. 2(b) in order for a clarified description of the thermal damage. The numbers in the nephograms in Fig. 2(b) show the coordinates of the locations. And the color level shows the value of thermal damage.

**Figure 3:**
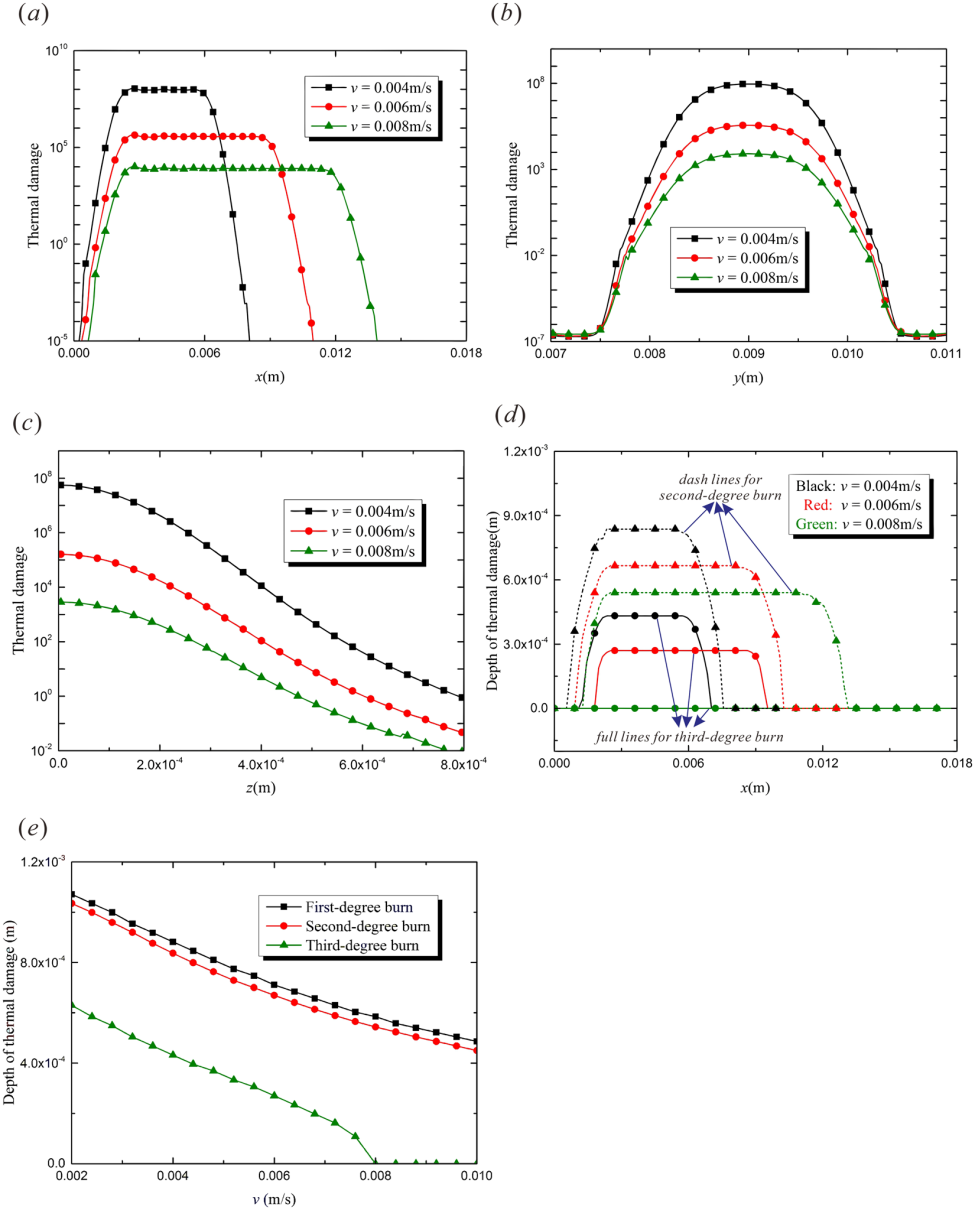
The effects of moving velocity of heat source, *v* ($t=1.5\,{\rm{s}}$, ${R}_{0}=0.002\,{\rm{m}}$, ${\tau }_{q}=0.05\,{\rm{s}}$, ${\tau }_{T}=0.5\,{\rm{s}}$). (**a**) Thermal damage variation with *x*-coordinate ($y=0.009\,{\rm{m}}$, $z=0$); (**b**) Thermal damage variation with *y*-coordinate ($x=0.0045\,{\rm{m}}$, $z=0$); (**c**) Thermal damage variation with *z*-coordinate ($y=0.009\,{\rm{m}}$, $x=0.0045\,{\rm{m}}$); (**d**) Depth of thermal damage variation with *x*-coordinate ($y=0.009\,{\rm{m}}$); (**e**) Depth of thermal damage varying with laser moving speed ($y=0.009\,{\rm{m}}$, $x=0.0045\,{\rm{m}}$).

It is obvious in Fig. 2 that the empyrosis occurs in the area subjected to the laser irradiation. The most severe burn appears along the center line of the affected area on the top surface, which agrees well with Fig. 3(b,c). The irradiation duration at the starting point of the path is shorter than the part behind, so the burn of the starting point is at a slight degree, which is also shown in Fig. 3(a,d).

Figure 3 shows the influences of moving velocity of heat source on the burn. The movement of the heating source brings about energy dispersion in the irradiated area^28,29^. The power input during a specific period is constant, so that a greater moving speed makes the laser energy spread in a broader area. Figure 3(a) shows the empyrosis variation on the laser moving path. The laser beam induces serious burn in the irradiated region. And the area of irradiated region is proportional to the moving velocity, with the double speed leading to the double area. However, the degree of thermal damage decreases because of the less energy concentration caused by the higher laser moving speed. Figure 3(b,c) show the thermal damage variation along the *y*- and *z*-coordinates, respectively. The heat-transfer capability of bio-tissue is weak, so that the width of the burnt region is just a little greater than the laser spot size *R*_0_. The laser moving speed has a great influence on the thermal damage in the irradiated region. The laser energy concentrates in a thin layer below the top surface, so the thermal damage maximizes on the top surface, namely $z=0$, and decreases rapidly with the increment of *z*. It can be found from Fig. 3(a–c) that the magnitude of empyrosis increases by hundreds of times with a thirty percent decrement of laser moving speed (from 0.006 m/s to 0.004 m/s). In order to visually demonstrate the effects of moving velocity on the degree of burn, the variations of the depth of second-degree (${\rm{\Omega }}=1$) and third-degree (${\rm{\Omega }}={10}^{4}$) thermal damage along the *x*-axis are shown in Fig. 3(d). The dashed lines show the second-degree burn while the solid lines for the third-degree burn. Figure 3(e) shows the depths of the three levels of burn vs the laser speed. It can be found from Fig. 3(d,e) that the minimum speed value causes the deepest burn wound. And the depth of thermal damage in the target tissue decreases with the increment of laser moving speed. The third-degree burn does not occur when the moving speed increases to *v* = 0.008 m/s. Since the heating power density is constant, the energy absorbed in the specified region increases as the moving speed of laser beam decreases. Consequently, the burn wound in this region will be more serious and deeper.

**Figure 4:**
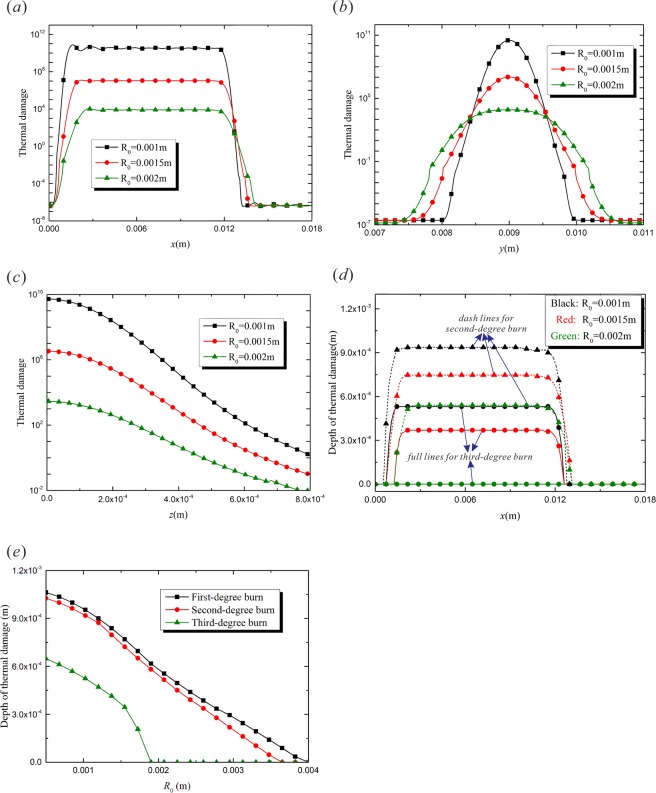
The effects of the laser spot size, *R*_*0*_ ($t=1.5\,{\rm{s}}$, $v=0.008\,{\rm{m}}/{\rm{s}}$, ${\tau }_{q}=0.05\,{\rm{s}}$, ${\tau }_{T}=0.5\,{\rm{s}}$). (**a**) Thermal damage variation with *x*-coordinate ($y=0.009\,{\rm{m}}$, $z=0$); (**b**) Thermal damage variation with *y*-coordinate ($x=0.0045\,{\rm{m}}$, $z=0$); (**c**) Thermal damage variation with *z*-coordinate ($y=0.009\,{\rm{m}}$, $x=0.0045\,{\rm{m}}$); (**d**) Depth of thermal damage variation with *x*-coordinate ($y=0.009\,{\rm{m}}$); (**e**) Depth of thermal damage varying with heat flux relaxation time ($y=0.009\,{\rm{m}}$, $x=0.0045\,{\rm{m}}$).

The laser spot size will affect the energy input per unit area. With a larger spot size, a wider region is irradiated by laser beam. Since the total laser energy power is constant, a specified position of the tissue absorbs less energy in such a case. That is to say, the energy dispersion caused by a bigger spot size will lead to a lower empyrosis degree. Figure 4(a) compares the variation of thermal damage along the laser moving path, which is induced by the laser beams with different spot sizes. It can be found that the spot size has significant influence on the burn injury. When the spot size decreases from ${R}_{0}=0.0015\,{\rm{m}}$ to ${R}_{0}=0.001\,{\rm{m}}$, the spotted area falls by 55%, while the value of Ω increases by one thousand times. Figure 4(b) shows the variation of burn injury along the *y*-axis, and it is shown that the maximum value of thermal damage shows up on the path of laser spot center. The decrement of the spot size leads to a significant increment of the peak value of ${\rm{\Omega }}$ and reduces the burnt area. The concentration of laser energy caused by the decreasing of the spot size also leads to higher-level burn in the depths of the target tissue, as is shown in Fig. 4(c). Figure 4(d,e) show the influences of laser spot size on the depth of different degrees of burn injury. It is shown that the depths of the three levels of burn decrease with the increment of laser spot size and the third-degree burn disappears when ${R}_{0}=0.002\,{\rm{m}}$.

It is known that the non-Fourier effect has significant influence on the empyrosis in the target bio-tissue. In the following, the influences of the two relaxation time ${\tau }_{q}$ and ${\tau }_{T}$ on the burn damage will be discussed.

**Figure 5:**
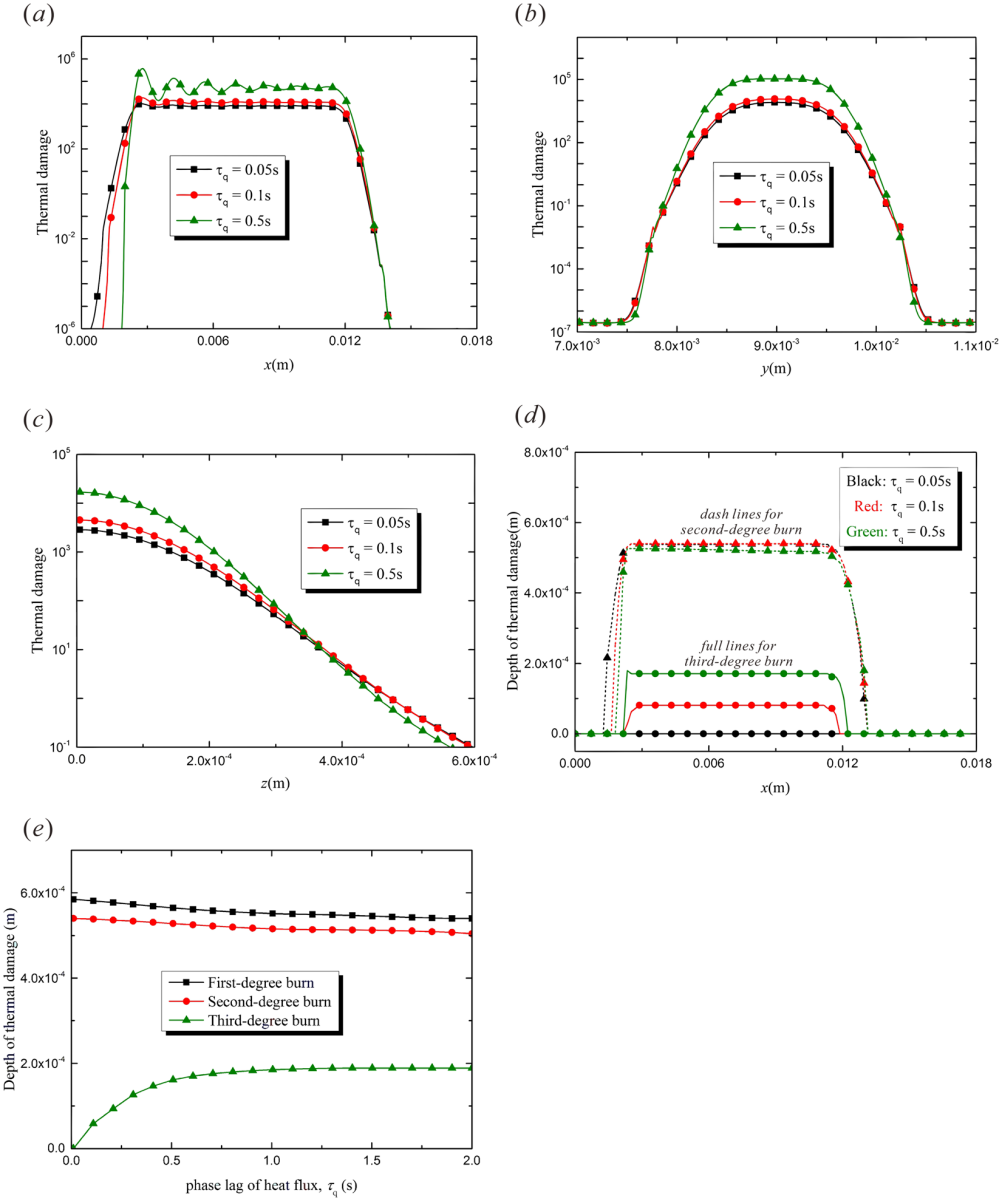
The effects of the relaxation time of heat flux, ${\tau }_{q}$ ($t=1.5\,{\rm{s}}$, ${R}_{0}=0.002\,{\rm{m}}$, $v=0.008\,{\rm{m}}/{\rm{s}}$, ${\tau }_{T}=0.5\,{\rm{s}}$). (**a**) Thermal damage variation with *x*-coordinate ($y=0.009\,{\rm{m}}$, $z=0$); (**b**) Thermal damage variation with *y*-coordinate ($x=0.0045\,{\rm{m}}$, $z=0$); (**c**) Thermal damage variation with *z*-coordinate ($y=0.009\,{\rm{m}}$, $x=0.0045\,{\rm{m}}$); (**d**) Depth of thermal damage variation with *x*-coordinate ($y=0.009\,{\rm{m}}$); (**e**) Depth of thermal damage varying with heat flux relaxation time ($y=0.009\,{\rm{m}}$, $x=0.0045\,{\rm{m}}$).

The heat flux relaxation ${\tau }_{q}$ means the heat flux lags behind the temperature variation. Consequently, the thermal energy propagation from the irradiated area to the surrounding tissue is arrested. A greater ${\tau }_{q}$ leads to more energy accumulated in the exposed region and a more remarkable vibration characteristics in thermal response^30^. Figure 5(a) shows the distribution of thermal damage along x-axis under different values of ${\tau }_{q}$. The fluctuation appears in the empyrosis distribution when ${\tau }_{q}$ increases from 0.05 s to 0.5 s. When *τ*_*q*_ is great enough, the fluctuant characteristics would appear in the temperature response, which has been reported in the existing literatures^22,31,32^. Since the thermal damage parameter Ω is determined by the integration of temperature function with regard to time, the fluctuation occurs as a result. The thermal damage in the affected region becomes more and more severe with the increasing heat accumulation caused by the growing of ${\tau }_{q}$. Moreover, the increment of ${\tau }_{q}$ also brings a smaller burn area, which is shown in Fig. 5(d). The variations of thermal damage along *y*- and *z*-directions are shown in Figs. 5(b,c). The thermal accumulation due to the delay of thermal energy propagation is remarkable. The increment of ${\tau }_{q}$ leads to an obvious rise of thermal damage in the irradiated region. Figure 5(d) shows the depth of burn injury varying along the laser moving path. When ${\tau }_{q}=0.05\,{\rm{s}}$, the retardation of the heat flux is not distinct, and the third-degree burn does not occur in the target tissue. On the other hand, when ${\tau }_{q}$ increases to 0.5 s, the hysteretic heat flux can not transfer the thermal energy on time. So more energy accumulates in the target region and the third-degree burn occurs in the depths of the tissue, as is shown by the solid lines in Fig. 5(d). The dashed lines show the depth of the second-degree burn. It is shown that the largest ${\tau }_{q}$ (${\tau }_{q}=0.5\,{\rm{s}}$) leads to the greatest depth of third-degree burn and the smallest depth of second-degree burn. However, the smallest ${\tau }_{q}$ (${\tau }_{q}=0.05\,{\rm{s}}$) causes the smallest depth of third-degree burn and the greatest depth of second-degree burn. This phenomenon agrees well with the conclusion that the phase lag of heat flux can arrest the energy transmission and cause the thermal energy concentration in the irradiated region^30^. Figure 5(e) shows the variation of the depth of thermal damage with ${\tau }_{q}$. It can be found that the depths of first- and second-degree burn are not sensitive to the value of ${\tau }_{q}$. However, the depth of the third-degree burn increases obviously with the increment of ${\tau }_{q}$, especially in the range of 0 ~ 0.5 s.

**Figure 6:**
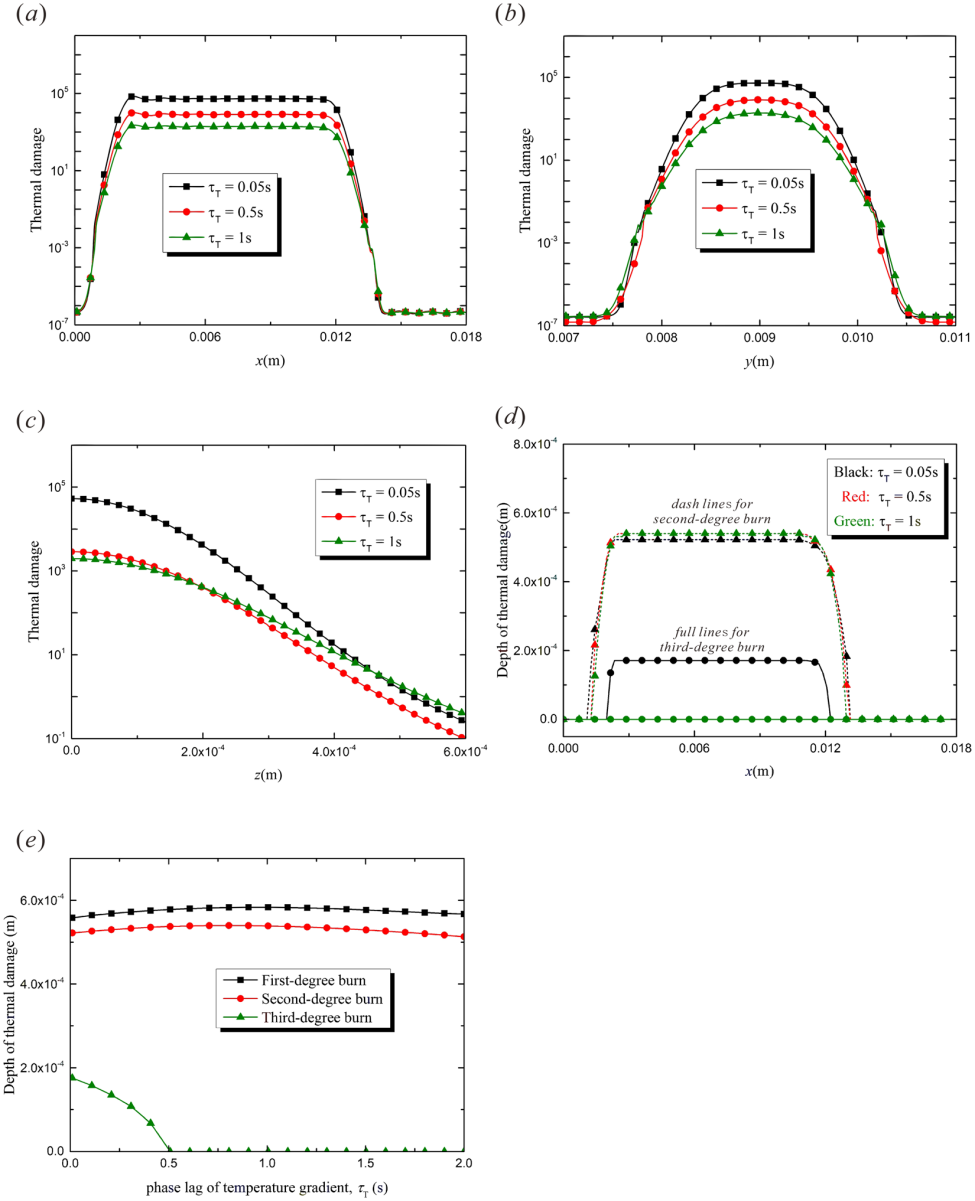
The effects of the relaxation time of temperature gradient, ${\tau }_{T}$ ($t=1.5\,{\rm{s}}$, ${R}_{0}=0.002\,{\rm{m}}$, ${\tau }_{q}=0.05\,{\rm{s}}$, $v=0.008\,{\rm{m}}/{\rm{s}}$). (**a**) Thermal damage variation with *x*-coordinate ($y=0.009\,{\rm{m}}$, $z=0$); (**b**) Thermal damage variation with *y*-coordinate ($x=0.0045\,{\rm{m}}$, $z=0$); (**c**) Thermal damage variation with *z*-coordinate ($y=0.009\,{\rm{m}}$, $x=0.0045\,{\rm{m}}$); (**d**) Depth of thermal damage variation with *x*-coordinate ($y=0.009\,{\rm{m}}$); (**e**) Depth of thermal damage varying with temperature relaxation time ($y=0.009\,{\rm{m}}$, $x=0.0045\,{\rm{m}}$).

The phase lag of temperature gradient, ${\tau }_{T}$, means that the heat flux precedes the temperature variation. The thermal transfer, which precedes the temperature increment, causes less energy accumulation and lower peak temperature in the irradiated area. The vibration characteristic in the thermal response recedes due to the increasing of ${\tau }_{T}$^30^. Figure 6(a,b) show the burn injury distribution along the laser moving path with different ${\tau }_{T}$. The increment of ${\tau }_{T}$ enhances the dispersion of the thermal energy, which means that the thermal damage is milder. Moreover, the thermal response area expands as ${\tau }_{T}$ increases. As a result, the phase lag of temperature gradient leads to milder burn in a wider affected region. Figure 6(c) shows the distribution of empyrosis in *z*-direction. The temperature retardation means that heat transfer occurs before the temperature response, so the heat accumulation in the surface layer of the target tissue is weakened and the peak value of thermal damage decreases. When ${\tau }_{T}=0.05\,{\rm{s}}$, the thermal damage, ${\rm{\Omega }}$, decreases rapidly along the thickness direction. However, when ${\tau }_{T}$ increases to 1 s, ${\rm{\Omega }}$ decreases more gradually. The obstruction of thermal accumulation leads to lower degree of burn and wider heat-affected zone, as is shown in Figs. 6(d,e). As ${\tau }_{T}$ increases, the depth of third-degree burn decreases obviously until it disappears when ${\tau }_{T}$ reaches 0.5 s. However, the regions of first- and second-degree burn are not sensitive to the variation of the temperature relaxation time ${\tau }_{T}$.

## Conclusions

Combined action of the non-Fourier effects, the metabolic thermal production and the heat diffusion by blood circulation makes the thermal transfer procedure in human tissue very perplexing. The present study explored the thermal damage distribution in a three-dimensional model of human tissue which is irradiated by a movable laser source. DPL biological thermal transfer model and Henriques’ thermal damage model were employed. The effects of the laser moving velocity, the laser spot size and the phase lags of heat flux and temperature gradient were discussed. The following conclusions can be drawn from the research:

The source moving velocity and the magnitude of laser spot influence the thermal damage by affecting the energy concentration degree. The laser moving speed mainly influences the thermal damage along the moving direction, namely the *x*-direction, however the effects of laser spot size show up on both *x*- and *y*-directions. Increment of the source moving velocity and the magnitude of laser spot makes the laser energy distributed over a much larger area and reduces the burn intensity level. With the comparison with spot magnitude, the source moving speed has greater influence on the burn depth.

The two thermal relaxation parameters, ${\tau }_{q}$ and ${\tau }_{T}$, also show distinct influences on the thermal damage. The phase lag of heat flux ${\tau }_{q}$ delays the thermal energy propagating from the irradiated region to the vicinity. Consequently, the thermal accumulation and burn degree increase with the increment of ${\tau }_{q}$. The depths of first- and second-degree burn reduce gently with ${\tau }_{q}$, but the depth of third-degree burn is more sensitive. On the contrary, the phase lag of temperature gradient ${\tau }_{T}$ delays the temperature response in the target tissue, implying the energy transmission occurs before the temperature varying. ${\tau }_{T}$ impedes the thermal accumulation and reduces the burn degree in the irradiated region. The depth of third-degree burn decreases obviously with the increasing of ${\tau }_{T}$.

## Data Availability

The raw/processed data required to reproduce these findings cannot be shared at this time due to technical or time limitations.
